# Aura and Stroke: relationship and what we have learnt from preclinical models

**DOI:** 10.1186/s10194-019-1016-x

**Published:** 2019-05-29

**Authors:** Muge Yemisci, Katharina Eikermann-Haerter

**Affiliations:** 1 Institute of Neurological Sciences and Psychiatry, and Faculty of Medicine, Department of Neurology, Hacettepe University, Ankara, Turkey; 2 Department of Radiology, Massachusetts General Hospital, Harvard Medical School, Boston, MA USA

**Keywords:** Migraine, Aura, Stroke, Spreading depolarization, Cerebrovascular disease, FHM, CADASIL, Pericyte, Microcirculation

## Abstract

**Background:**

Population-based studies have highlighted a close relationship between migraine and stroke. Migraine, especially with aura, is a risk factor for both ischemic and hemorrhagic stroke. Interestingly, stroke risk is highest for migraineurs who are young and otherwise healthy.

**Main body:**

Preclinical models have provided us with possible mechanisms to explain the increased vulnerability of migraineurs’ brains towards ischemia and suggest a key role for enhanced cerebral excitability and increased incidence of microembolic events. Spreading depolarization (SD), a slowly propagating wave of neuronal depolarization, is the electrophysiologic event underlying migraine aura and a known headache trigger. Increased SD susceptibility has been demonstrated in migraine animal models, including transgenic mice carrying human mutations for the migraine-associated syndrome CADASIL and familial hemiplegic migraine (type 1 and 2). Upon experimentally induced SD, these mice develop aura-like neurological symptoms, akin to patients with the respective mutations. Migraine mutant mice also exhibit an increased frequency of ischemia-triggered SDs upon experimental stroke, associated with accelerated infarct growth and worse outcomes. The severe stroke phenotype can be explained by SD-related downstream events that exacerbate the metabolic mismatch, including pericyte contraction and neuroglial inflammation. Pharmacological suppression of the genetically enhanced SD susceptibility normalizes the stroke phenotype in familial hemiplegic migraine mutant mice. Recent epidemiologic and imaging studies suggest that these preclinical findings can be extrapolated to migraine patients. Migraine patients are at risk for particularly cardioembolic stroke. At the same time, studies suggest an increased incidence of coagulopathy, atrial fibrillation and patent foramen ovale among migraineurs, providing a possible path for microembolic induction of SD and, in rare instances, stroke in hyperexcitable brains. Indeed, recent imaging studies document an accelerated infarct progression with only little potentially salvageable brain tissue in acute stroke patients with a migraine history, suggesting an increased vulnerability towards cerebral ischemia.

**Conclusion:**

Preclinical models suggest a key role for enhanced SD susceptibility and microembolization to explain both the occurrence of migraine attacks and the increased stroke risk in migraineurs. Therapeutic targeting of SD and microembolic events, or potential causes thereof, will be promising for treatment of aura and may also prevent ischemic infarction in vulnerable brains.

## Background

### Migraine and Aura

Migraine is a chronic or episodic neurological disorder that is typically characterized by throbbing or pulsatile unilateral headaches lasting for 4–72 h. The high prevalence of migraine and the resulting disability places migraine among the top diseases culminating in a high socioeconomic burden. Thirty percent of migraineurs develop transient neurological symptoms in the setting of an attack, the so-called migraine aura [1]. Aura symptoms characteristically occur up to 1 h prior to the headache, but sometimes can overlap with the headache phase. At least two migraine attacks preceded by an aura are needed to establish the diagnosis of migraine with aura (MA). The clinical characteristics of migraine aura included in the formal migraine classification [2] International Classification of Headache Disorders (ICHD-3) are visual, sensory, language, or motor symptoms as well as brainstem symptoms [1]. Visual symptoms are the most commonly encountered aura feature. Brainstem aura symptoms are rare but particularly common in familial hemiplegic migraine [1, 3].

Since the initial description by Leão in 1944 [4], spreading depolarization (SD) has been recognized as the electrophysiological correlate of migraine aura. SD is characterized by prompt, self-propagating neuronal depolarization waves that spread at a speed of 3–5 mm/min [5, 6]. Strongest evidence for a key role of SD in migraine aura comes from a functional MRI study that showed retinotopic congruence between the visual aura perception and SD-typical BOLD signal changes traversing the occipital cortex [7]. Preclinical studies in transgenic mice for familial hemiplegic migraine (FHM) further underscore a key role for SD in migraine pathophysiology. FHM is an autosomal dominant severe migraine subtype, with associated hemiplegic aura preceding some attacks. FHM has a prevalence of 5/100000. Three FHM mutations have been identified so far; the mutations are found in voltage dependent, P/Q type calcium channel alpha 1A subunit CACNA1A for FHM1; ATPase, Na+/K+ transporting, alpha 2 polypeptide ATP1A2 for FHM2; and sodium channel, voltage gated, type 1 alpha subunit SCN1A for FHM3 [8, 9]. In transgenic mice carrying human mutations for FHM type 1, experimental induction of SD produces migraine-aura like symptoms. Mice with the R192Q mutation develop transient hemiplegia, whereas mice with the severe S218 L mutation also develop seizures, similar to the clinical phenotype in patients with the respective mutation [10]. These severe aura symptoms were associated with a facilitated subcortical spread of SD [11]. Upon exposure to an SD trigger (KCl or electrical stimulation), FHM1 mice show a reduced threshold for SD induction and develop a higher number of SDs, with S218 L mice carrying the stronger gain-of-function mutation exhibiting a more severe SD phenotype when compared to R192Q mice [10]. This enhanced SD susceptibility seems to be related to stronger cortical synapses, as indicated by larger axonal boutons and an increased percentage of highly excitable mushroom type dendritic spines with a high number of excitatory NMDA receptors [12]. Interestingly, SD susceptibility is further increased in female mice compared to male FHM1 mice, consistent with an increased migraine incidence in females compared to males. Hormonal ablation in FHM1 mice successfully abrogated the gender difference in SD susceptibility [13], underscoring the importance of sex hormones in further modulating the genetically enhanced SD susceptibility. An increased SD susceptibility has also been demonstrated in transgenic mice for FHM type 2 [14], as well as familial migraine and advanced sleep phase [15]. Importantly, SD can be induced by microembolic events [16], and even occlusion of a single cortical arteriole is sufficient to trigger SD [17], providing a candidate mechanism for SD induction in the migraine-susceptible brain. SD also plays a major role in other diseases, and worsens outcomes in ischemic stroke, intracranial hemorrhage, traumatic brain injury and subarachnoid hemorrhage [18, 19].

### Migraine and stroke

The World Health Organization lists stroke as the second leading cause of death and the third leading cause of serious long-lasting disability [20]. Recent reports have highlighted the fact that 90% of strokes are preventable, which reflects an opportunity to decrease stroke related mortality and morbidity [21]. Strategies primarily target modifiable vascular risk factors such as hypertension, hyperlipidemia, diabetes and smoking [22]. Over the years, data accumulating from experimental and clinical studies have pointed out an important role for migraine as another potentially modifiable risk factor contributing to the stroke burden [23–26]. The association between migraine and stroke, both of which are considered as multifaceted neurovascular disorders, is especially pronounced in young female patients with no other stroke risk factors; overall, a history of migraine doubles the risk of stroke [23–26]. Importantly, the stroke risk is higher in migraineurs with aura compared to those without aura.

The relationship between migraine and stroke has been studied for years, both experimentally and clinically. Numerous theories have been proposed, involving a shared genetic basis, vascular dysfunction, patent foramen ovale (PFO), atrial fibrillation, increased inflammation and excitotoxicity, as well as abnormally increased coagulation [18, 27–36]. Twin studies suggest a contribution of familial factors to underlie the migraine stroke association [37], and a genome-wide analysis of common variants has identified a shared genetic susceptibility to migraine and ischemic stroke [34]. Preclinical studies have confirmed an increased cerebral vulnerability to ischemia in transgenic mice carrying human migraine mutations. In FHM1 transgenic mice, occlusion of the middle cerebral artery causes an increased number of ischemia-triggered SDs with facilitated initiation of anoxic depolarization, known to exacerbate the metabolic mismatch and worsen infarcts. Accordingly, diffusion weighted MRI documents an accelerated expansion of the infarct core in migraine mutant mice, with only a small amount of potentially salvageable brain tissue, the so-called penumbra [8]. In fact, high-frequent ischemic depolarizations have been shown to adversely affect tissue and neurological outcomes in the setting of cerebral ischemia even in wild-type mice [38]. Accordingly, migraine prophylactic drugs that suppress the genetically increased SD susceptibility in FHM mutant mice reduce the number of ischemia-triggered SDs upon experimental middle cerebral artery occlusion, and improve, even normalize, the severe stroke phenotype [39]. Another autosomal dominant rare migraine-associated disease is cerebral autosomal dominant arteriopathy with subcortical infarcts and leukoencephalopathy (CADASIL). CADASIL is caused by mutations in the *NOTCH3* gene and is characterized by vasculopathy in perforator cerebral arteries secondary to fibrosis and accumulation of osmiophilic substances [40]. Migraine with aura is generally the first symptom and found in 30–40% of CADASIL patients. At later stages, ischemic stroke develops in some patients, while migraine attacks lessen or even stop [2, 40]. Patients typically show a reduced vascular smooth muscle cell function/relaxation [41]. Similarly, transgenic mice expressing the human Notch 3 R90C mutation as well as Notch 3 knockout mice develop arterial pathological hallmarks of CADASIL as well as cerebrovascular dysfunction, and show an enhanced susceptibility to SD [9, 11]. Upon experimental middle cerebral artery occlusion, stroke sizes are enlarged with an increased frequency of ischemia-triggered spreading depolarizations, and neurological outcomes are worse when compared to wild-type littermates [42]. These experimental data suggest enhanced SD susceptibility to be a key factor for the increased stroke risk in migraine-susceptible brains. Along the same line, factors increasing the likelihood of SD occurrence, or “endogenous” SD trigger factors, seem increased in migraineurs. For example, genetic and epidemiologic studies document an increased incidence of hypercoagulability [43], persistent foramen ovale [44], and atrial fibrillation [36] among migraineurs, which facilitate microembolic events in cerebral vasculature as triggers for SD, migraine and possibly stroke.

## Mechanisms underlying the migraine, SD and Stroke Association

Consistent with the accelerated infarct growth in migraine mutant mice, acute stroke patients with a history of migraine also show rapid infarct expansion. There is only a small amount of potentially salvageable brain tissue/mismatch in migraineurs, when penumbra is determined by cerebral blood volume (CBV) / mean transit time (MTT) mismatch on CT perfusion [45], or diffusion weighted imaging (DWI) / MTT mismatch on MR perfusion [46]. Additionally, the amount of penumbra that could be salvaged was smaller among MA patients in comparison to MO and non-migraineous cases, highlighting increased brain tissue vulnerability in migraineurs [45]. Preclinical data support a key role for SD susceptibility in mediating stroke risk in migraineurs. In fact, as outlined below in more detail, there is evidence in migraine-susceptible brains for an increased incidence of 1) SD triggering factors such as microemboli, 2) a reduced threshold for SD induction, 3) an increased frequency of ischemia-triggered SD, and 4) worse consequences of individual SDs on the metabolic mismatch. However, considering that migraineurs also have an increased risk of myocardial infarction, venous thromboembolism and atrial fibrillation, a systemic dysfunction not limited to the cerebral vasculature could also contribute to the increased stroke risk in migraineurs [36, 47, 48].

### Increased SD susceptibility and its consequences

Preclinical and imaging studies highlight enhanced SD susceptibility as a candidate mechanism increasing brain vulnerability to ischemia and thereby contributing to the stroke risk in migraineurs. These findings have paved the way for studies focusing on the consequences of SD, and thereby migraine, on ischemic stroke. Following SD, cerebral blood flow is reduced for hours, after a brief initial functional hyperemia, with an associated decrease in induced neuronal and glial calcium responses [49, 50]. Even a single episode of SD in rats is associated with a long-lasting rise in the cerebral metabolic rate of oxygen, a reduction in cerebral blood flow and impaired neurovascular coupling [49]. Migraineurs’ brains might even be more vulnerable to the negative and sometimes long-lasting effects of SD on microvasculature, leading to an accumulation of pathological cellular changes secondary to repetitive ischemic events in the long run [5, 6, 51]. In fact, SD-related changes in neuronal calcium levels and transient hypoxia are more severe in FHM transgenic mice when compared to wild-type mice, suggesting that the consequences of SD are more pronounced in migraine-susceptible brains [12, 50]. Therefore, cerebral hypoperfusion that remains unnoticed in the non-migraneur’s brain might cause profound ischemic lesions, and in rare instances even clinically manifest stroke in a migraine-susceptible brain. Indeed, white matter abnormalities, infarct-like lesions as well as volumetric changes in gray and white matter were found to be more frequent in migraineurs, particularly with aura, when compared to controls [52–54].

SD-typical dynamic changes in cerebral blood flow are attributed to an impaired vascular reactivity of cortical vessels [55]. In fact, SD impairs vascular smooth muscle function as evidenced by a reduced vasodilatory response of isolated rat middle cerebral artery to extraluminal acidosis, and an increased vascular reaction to extraluminal K^+^ [55]. Similar observations have been made in migraine patients [56]. Migraineurs exhibit an increased cerebrovascular reactivity to hypocapnia and decreased vascular reactivity to vasodilatory agents such as acetazolamide or L-arginine, pointing to a reduced tone of cerebral vessels and/or endothelial dysfunction [57, 58]. These findings suggest a reduced vasomotor reserve in cerebral microvasculature in migraineurs, and recent studies suggest a key role for pericytes to underlie this altered cerebrovascular reactivity.

### Role for Pericytes and autoregulation in mediating the effects of SD

Pericytes are contractile mural cells in the cerebral and retinal vasculature that express alpha-smooth muscle actin and cover capillaries [59–61]. As a critical component of the neurovascular unit, pericytes have an important role in regulating microcirculation via constriction and relaxation, thereby controlling local cerebral blood flow in physiological and disease states such as stroke [62–68]. In the setting of cerebral ischemia, prolonged constriction of pericytes might lead to microvascular occlusion and unfavorable outcomes [69, 70]. Recently, it has been shown that capillary pericytes also have an active role in the regulation of cortical blood vessels during and after SD [71]. In fact, SD-induced prolonged vasoconstriction is strongest in first order capillaries with a persistent increase in pericyte calcium. Following SD, somatosensory stimulation fails to evoke further changes in capillary diameter and pericyte calcium, suggesting a key role for pericytes in mediating long-lasting oligemia following SD [71]. Recent studies in Notch3 transgenic mice indeed revealed a loss of pericytes with reduced coverage of capillaries and Notch3 aggregations around the few remaining pericytes which might explain microcirculatory dysfunction and ischemia in CADASIL mutant mice and patients [72–75]. In fact, CADASIL mutant mice show blood brain barrier leakage, reduced vasomotor reactivity to CO_2_, and narrowing or occlusion of microvessels, which may result from reduced pericyte function [74, 75]. Similarly, in patients with CADASIL, resting cerebral blood flow and vasodilatory response are reduced while there is an increase in oxygen extraction fraction and endothelial dysfunction [76–79]. And just very recently, structural changes involving pericytes and endothelial cells of microvessels have also been identified in FHM patients [80]. Therefore, prevention or treatment of pericyte constriction may become a therapeutic target in MA and migraine-related cerebral ischemia [71, 81].

The potent vasodilator calcitonin gene-related peptide (CGRP), and pharmacological suppression thereof, may further modulate the altered cerebrovascular autoregulation and the increased vulnerability to cerebral ischemia in migraineurs. CGRP receptors are present in both the nervous and cardiovascular system, underscoring a significant role for CGRP in regulating vascular resistance and regional blood flow in cerebral health and disease [82]. In fact, endogenous CGRP is protective against neuronal damage in the setting of acute or chronic stroke, as suggested by experiments using CGRP knockout mice. CGRP has been shown to reduce infarct size [83], and CGRP is protective against cerebral vasospasm in the setting of subarachnoid hemorrhage [84]. CGRP might also be protective in individuals with chronic bilateral carotid stenosis by reducing subsequent neuronal injury and cognitive impairment [83]. The protective role of CGRP in the setting of cerebral ischemia may be particularly relevant in patients with migraine for the following reasons. During migraine headache, activation of the trigeminovascular system triggers the release of CGRP from trigeminal sensory nerves [85–87], and stimulation of sensory fibers has been shown to increase CGRP with consecutive dilatation of cerebral and dural vessels [88]. Accordingly, CGRP was found to be elevated in external jugular venous blood samples of migraine patients during migraine attacks [89]. In turn, intravenous infusion of CGRP has been shown to cause attacks in some migraine patients [90]. Because CGRP may act as a vasodilatory safeguard during cerebral ischemic events in migraine patients, pharmacological CGRP blockade, efficacious as acute and preventive treatment of migraine [86, 91], may exacerbate the increased stroke risk in migraineurs. Antibodies against CGRP or its receptor may further enhance vulnerability to cerebral ischemia in migraineurs, with the risk of transient mild ischemic events progressing to an ischemic stroke [92]. However, no safety issues suggesting a cerebrovascular risk for anti-CGRP drugs have emerged from clinical trials so far, with possible long-term effects still not sufficiently investigated [93].

### Role for Neuroinflammation in mediating the effects of SD

Neuroinflammation contributes to ischemic complications related to migraine. Inflammatory cascades are involved in the detrimental effects of SD in migraine and stroke. Animal studies showed that SD induces neuronal and glial release of inflammatory mediators, dural mast cell degranulation as well as activation the trigeminovascular system [94–97]. Within minutes after SD, the neuronal hemichannel pannexin 1 opens and forms a pore complex with the ligand-gated cation channel P2X7, allowing the release of excitatory neurotransmitters to sustain SD and activate neuroinflammation [96]. Inhibition of SD-induced opening of the neuronal Pannexin1 megachannel suppresses SD and reduces SD-induced inflammatory downstream cascades that might lead to headache [97], including upregulation of interleukin-1 beta (IL-1beta), inducible nitric oxide synthase and cyclooxygenase-2 in the cortex. IL-1beta as a pro-inflammatory cytokine in rat trigeminal ganglia cells causes the release of prostaglandin E2/CGRP and induces the activation of meningeal nociceptors, mechanically sensitizing and activating nociceptors that innervate the intracranial meninges and possibly involved in initiating throbbing headache [98–101]. Accordingly, pore-inhibitors also suppress surrogates for trigeminovascular activation, including the expression of calcitonin gene-related peptide in the trigeminal ganglion and c-Fos in the trigeminal nucleus caudalis [97]. Therefore, inhibition of neuroinflammation might be protective in both migraine and stroke by suppressing SD and direct cellular damage in the setting of ischemia [96, 97].

### Increased SD triggers in Migraineurs

Cerebral microembolization may induce SD and thereby trigger a migraine attack. In mice, different types of microemboli injected through the carotid artery, mimicking embolization in humans, were found to induce SD [16]. Embolic occlusion of even a single penetrating artery imitating cerebral microembolism has been shown to induce SD, leading to selective neuronal death and small infarction [17]. Similarly, thrombotic occlusion of a single ascending cortical vein can also induce SD, albeit less frequently [102–104]. These microembolic events might contribute to long-lasting hypoperfusion if they are repetitive and if there is a predisposing condition like MA, with increased vulnerability to cerebral ischemia [40]. Microembolic small vessel occlusion in the setting of persistent foramen ovale or silent vessel dissections that remain completely unnoticed in non-migrainous brains might therefore lead to SD and ischemic complications or even infarct in migraine susceptible brains. This hypothesis is consistent with an enhanced likelihood of clot formation in migraineurs, who reportedly show an increased incidence of hypercoagulability [43] and atrial fibrillation [36]. As a potential path for cerebral microembolism, an increased incidence of persistent foramen ovale has been reported in migraineurs [105], and paradoxical air microembolism as well as cardiac catheterization with presumably associated microembolic events have been shown to induce headache in migraineurs [106–108]. Along the same line, the increased stroke risk in migraineurs is highest for stroke of cardioembolic etiology, and in the peri-operative emboli-prone setting [109, 110].

## Conclusions

**Fig. 1:**
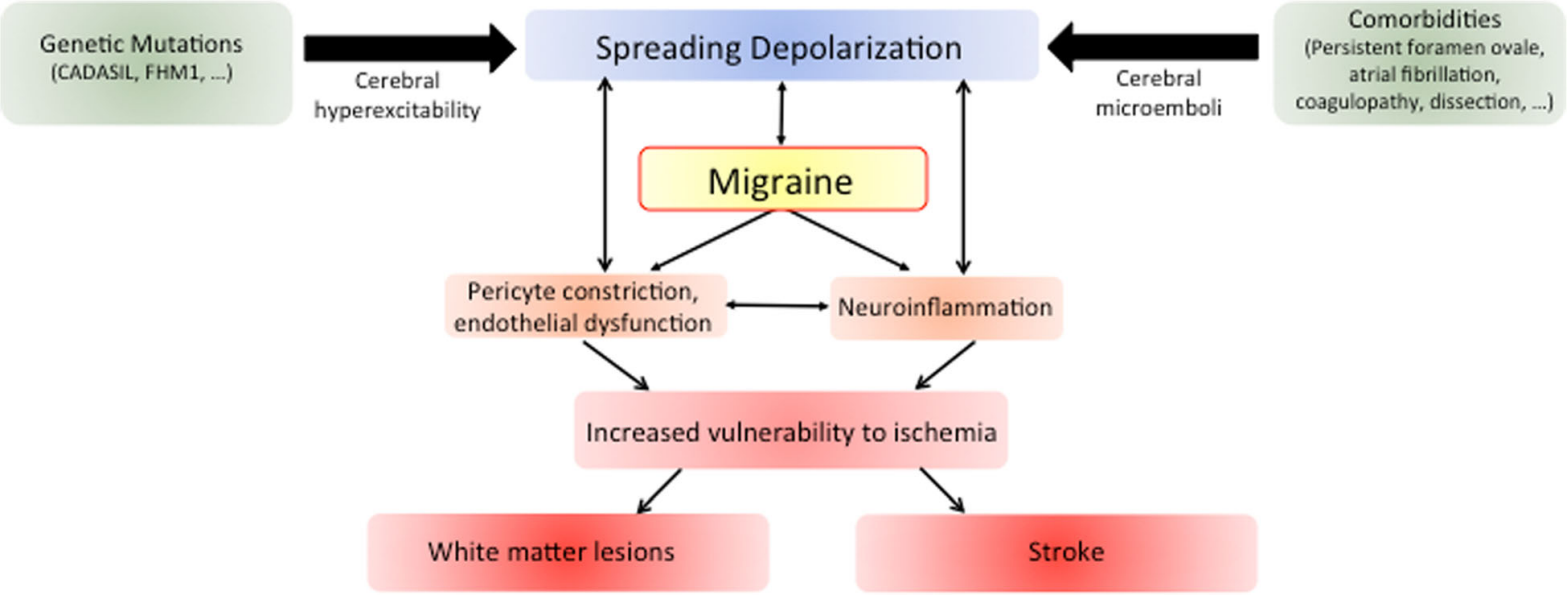
The interplay between migraine and stroke. Shared genetic factors and associated clinical features commonly observed in migraine patients contribute to the link between migraine and stroke. The underlying mechanism involves facilitation of spreading depolarization (SD), the electrophysiological correlate of aura, via increased SD trigger factors and/or reduced threshold for SD induction. SD then causes migraine as well as neuroinflammation and vascular dysfunction, increasing the brain’s vulnerability to ischemia. This cascade may result in clinically silent ischemic lesions that are frequently observed in migraineurs, or even cause ischemic stroke

Studies suggest a double-hit hypothesis linking migraine with stroke (Fig. 1). In the setting of a genetically enhanced cerebral excitability, microembolization and/or inflammatory mediators among other factors might trigger SD, which causes or exacerbates focal ischemia; these insults remain transient or asymptomatic in most instances. However, in the setting of a vulnerable/hyperexcitable brain, these perturbations might be more severe or prolonged, escalating into further downstream events such as silent ischemic lesions or even ischemic stroke.

## Data Availability

Not applicable.

## Abbreviations

CADASIL: Cerebral autosomal dominant arteriopathy with subcortical infarcts and leukoencephalopathy
GWAS: Genome-wide association study
ICHD: International Classification of Headache Disorders
KCl: Potassium chloride
MA: Migraine with aura
MO: Migraine without aura
MRI: Magnetic resonance imaging
PFO: Patent foramen ovale
SD: Spreading depolarization

## References

[1] Russell MB, Olesen J (1995). Increased familial risk and evidence of genetic factor in migraine. BMJ.

[2] Headache Classification Committee of the International Headache Society (IHS) (2018). The international classification of headache disorders, 3rd edition. Cephalalgia.

[3] Thomsen LL, Eriksen MK, Roemer SF, Andersen I, Olesen J, Russell MB (2002). A population-based study of familial hemiplegic migraine suggests revised diagnostic criteria. Brain..

[4] Leão AAP (1944). Spreading depression of activity in the cerebral cortex. J Neurophysiol.

[5] Somjen GG (2001). Mechanisms of spreading depression and hypoxic spreading depression-like depolarization. Physiol Rev.

[6] Dreier JP (2011). The role of spreading depression, spreading depolarization and spreading ischemia in neurological disease. Nat Med.

[7] Hadjikhani N, Sanchez Del Rio M, Wu O, Schwartz D, Bakker D, Fischl B, Kwong KK, Cutrer FM, Rosen BR, Tootell RB, Sorensen AG, Moskowitz MA (2001). Mechanisms of migraine aura revealed by functional MRI in human visual cortex. Proc Natl Acad Sci U S A.

[8] Eikermann-Haerter K, Lee JH, Yuzawa I, Liu CH, Zhou Z, Shin HK, Zheng Y, Qin T, Kurth T, Waeber C, Ferrari MD, van den Maagdenberg AM, Moskowitz MA, Ayata C (2012). Migraine mutations increase stroke vulnerability by facilitating ischemic depolarizations. Circulation..

[9] Chen SP, Tolner EA, Eikermann-Haerter K (2016). Animal models of monogenic migraine. Cephalalgia..

[10] Eikermann-Haerter K, Dileköz E, Kudo C, Savitz SI, Waeber C, Baum MJ, Ferrari MD, van den Maagdenberg AM, Moskowitz MA, Ayata C (2009). Genetic and hormonal factors modulate spreading depression and transient hemiparesis in mouse models of familial hemiplegic migraine type 1. J Clin Invest.

[11] Eikermann-Haerter K, Yuzawa I, Qin T, Wang Y, Baek K, Kim YR, Hoffmann U, Dilekoz E, Waeber C, Ferrari MD, van den Maagdenberg AM, Moskowitz MA, Ayata C (2011). Enhanced subcortical spreading depression in familial hemiplegic migraine type 1 mutant mice. J Neurosci.

[12] Eikermann-Haerter Katharina, Arbel-Ornath Michal, Yalcin Nilufer, Yu Esther S., Kuchibhotla Kishore V., Yuzawa Izumi, Hudry Eloise, Willard Carli R., Climov Mihail, Keles Fatmagul, Belcher Arianna M., Sengul Buse, Negro Andrea, Rosen Isaac A., Arreguin Andrea, Ferrari Michel D., van den Maagdenberg Arn M. J. M., Bacskai Brian J., Ayata Cenk (2015). Abnormal synaptic Ca2+homeostasis and morphology in cortical neurons of familial hemiplegic migraine type 1 mutant mice. Annals of Neurology.

[13] Eikermann-Haerter K, Baum MJ, Ferrari MD, van den Maagdenberg AM, Moskowitz MA, Ayata C (2009). Androgenic suppression of spreading depression in familial hemiplegic migraine type 1 mutant mice. Ann Neurol.

[14] Kros L, Lykke-Hartmann K, Khodakhah K (2018). Increased susceptibility to cortical spreading depression and epileptiform activity in a mouse model for FHM2. Sci Rep.

[15] Brennan KC, Bates EA, Shapiro RE, Zyuzin J, Hallows WC, Huang Y, Lee HY, Jones CR, Fu YH, Charles AC, Ptáček LJ (2013). Casein kinase iδ mutations in familial migraine and advanced sleep phase. Sci Transl Med.

[16] Nozari A, Dilekoz E, Sukhotinsky I, Stein T, Eikermann-Haerter K, Liu C, Wang Y, Frosch MP, Waeber C, Ayata C, Moskowitz MA (2010). Microemboli may link spreading depression, migraine aura, and patent foramen ovale. Ann Neurol.

[17] Donmez-Demir B, Yemisci M, Kilic K, Gursoy-Ozdemir Y, Soylemezoglu F, Moskowitz M, Dalkara T (2018). Microembolism of single cortical arterioles can induce spreading depression and ischemic injury; a potential trigger for migraine and related MRI lesions. Brain Res.

[18] Ayata C, Lauritzen M (2015). Spreading depression, spreading depolarizations, and the cerebral vasculature. Physiol Rev.

[19] Lauritzen M, Strong AJ (2017). Spreading depression of Leão' and its emerging relevance to acute brain injury in humans. J Cereb Blood Flow Metab.

[20] Johnson W, Onuma O, Owolabi M, Sachdev S (2016). Stroke: a global response is needed. Bull World Health Organ.

[21] O'Donnell MJ, Xavier D, Liu L, Zhang H, Chin SL, Rao-Melacini P, Rangarajan S, Islam S, Pais P, MJ MQ, Mondo C, Damasceno A, Lopez-Jaramillo P, Hankey GJ, Dans AL, Yusoff K, Truelsen T, Diener HC, Sacco RL, Ryglewicz D, Czlonkowska A, Weimar C, Wang X, Yusuf S, INTERSTROKE investigators (2010). Risk factors for ischaemic and intracerebral haemorrhagic stroke in 22 countries (the INTERSTROKE study): a case-control study. Lancet.

[22] Johnston SC, Mendis S, Mathers CD (2009). Global variation in stroke burden and mortality: estimates from monitoring, surveillance, and modelling. Lancet Neurol.

[23] Chang CL, Donaghy M, Poulter N (1999). Migraine and stroke in young women: case-control study. The World Health Organisation collaborative study of cardiovascular disease and steroid hormone contraception. BMJ..

[24] Kurth T, Schurks M, Logroscino G, Buring JE (2009). Migraine frequency and risk of cardiovascular disease in women. Neurology..

[25] Spector JT, Kahn SR, Jones MR, Jayakumar M, Dalal D, Nazarian S (2010). Migraine headache and ischemic stroke risk: an updated meta-analysis. Am J Med.

[26] Kurth T, Chabriat H, Bousser MG (2012). Migraine and stroke: a complex association with clinical implications. Lancet Neurol.

[27] Tietjen GE (2007). Migraine and ischaemic heart disease and stroke: potential mechanisms and treatment implications. Cephalalgia..

[28] Tietjen GE (2007). Migraine as a systemic disorder. Neurology..

[29] Stam AH, Haan J, van den Maagdenberg AM, Ferrari MD, Terwindt GM (2009). Migraine and genetic and acquired vasculopathies. Cephalalgia..

[30] Eikermann-Haerter K, Ayata C (2010). Cortical spreading depression and migraine. Curr Neurol Neurosci Rep..

[31] Eikermann-Haerter K (2014). Spreading depolarization may link migraine and stroke. Headache..

[32] Kazemi H, Speckmann EJ, Gorji A (2014). Familial hemiplegic migraine and spreading depression. Iran J Child Neurol.

[33] Sacco S, Kurth T (2014). Migraine and the risk for stroke and cardiovascular disease. Curr Cardiol Rep.

[34] Malik R, Freilinger T, Bendik S, Winsvold BS, Anttila V, Vander Heiden J (2015). Shared genetic basis for migraine and ischemic stroke. A genome-wide analysis of common variants. Neurology..

[35] Eikermann-Haerter K, Yuzawa I, Dilekoz E, Joutel A, Moskowitz MA, Ayata C (2011). Cerebral autosomal dominant arteriopathy with subcortical infarcts and leukoencephalopathy syndrome mutations increase susceptibility to spreading depression. Ann Neurol.

[36] Sen S, Androulakis XM, Duda V, Alonso A, Chen LY, Soliman EZ, Magnani J, Trivedi T, Merchant AT, Gottesman RF, Rosamond WD (2018). Migraine with visual aura is a risk factor for incident atrial fibrillation: A cohort study. Neurology.

[37] Lantz M, Sieurin J, Sjölander A, Waldenlind E, Sjöstrand C, Wirdefeldt K (2017). Migraine and risk of stroke: a national population-based twin study. Brain..

[38] Shin HK, Dunn AK, Jones PB, Boas DA, Moskowitz MA, Ayata C (2006). Vasoconstrictive neurovascular coupling during focal ischemic depolarizations. J Cereb Blood Flow Metab.

[39] Eikermann-Haerter K, Lee JH, Yalcin N, Yu ES, Daneshmand A, Wei Y, Zheng Y, Can A, Sengul B, Ferrari MD, van den Maagdenberg AM, Ayata C (2015). Migraine prophylaxis, ischemic depolarizations, and stroke outcomes in mice. Stroke..

[40] Gladstone JP, Dodick DW (2005). Migraine and cerebral white matter lesions: when to suspect cerebral autosomal dominant arteriopathy with subcortical infarcts and leukoencephalopathy (CADASIL). Neurologist..

[41] de Boer I, Stam AH, Buntinx L, Zielman R, van der Steen I, van den Maagdenberg AMJM, de Koning EJP, Ferrari MD, de Hoon JN, Terwindt GM (2018). RVCL-S and CADASIL display distinct impaired vascular function. Neurology..

[42] Lee JH, Eikermann-Haerter K, Joutel A, Moskowitz MA, Ayata C (2009) Enlarged infarcts in mice expressing the archetypal Notch3 R90C CADASIL mutation. J Cereb Blood Flow Metab 29:S253-S254

[43] Tietjen GE, Collins SA (2018). Hypercoagulability and migraine. Headache..

[44] Wilmshurst PT (2018). Migraine with aura and persistent foramen ovale. Eye (Lond).

[45] Pezzini A, Busto G, Zedde M, Gamba M, Zini A, Poli L, Caria F, De Giuli V, Simone AM, Pascarella R, Padovani A, Padroni M, Gasparotti R, Colagrande S, Fainardi E (2018). Vulnerability to infarction during cerebral ischemia in migraine sufferers. Stroke..

[46] Mawet J, Eikermann-Haerter K, Park KY, Helenius J, Daneshmand A, Pearlman L, Avery R, Negro A, Velioglu M, Arsava EM, Ay H, Ayata C (2015). Sensitivity to acute cerebral ischemic injury in migraineurs: a retrospective case-control study. Neurology..

[47] Bigal ME, Kurth T, Santanello N, Buse D, Golden W, Robbins M, Lipton RB (2010). Migraine and cardiovascular disease: a population-based study. Neurology..

[48] Adelborg K, Szépligeti SK, Holland-Bill L, Ehrenstein V, Horváth-Puhó E, Henderson VW, Sørensen HT (2018). Migraine and risk of cardiovascular diseases: Danish population based matched cohort study. BMJ..

[49] Piilgaard H, Lauritzen M (2009). Persistent increase in oxygen consumption and impaired neurovascular coupling after spreading depression in rat neocortex. J Cereb Blood Flow Metab.

[50] Khennouf Lila, Gesslein Bodil, Lind Barbara Lykke, van den Maagdenberg Arn M.J.M., Lauritzen Martin (2016). Activity-dependent calcium, oxygen, and vascular responses in a mouse model of familial hemiplegic migraine type 1. Annals of Neurology.

[51] Gryglas A, Smigiel R (2017). Migraine and stroke: What's the link? What to do?. Curr Neurol Neurosci Rep.

[52] Kruit MC, van Buchem MA, Hofman PA, Bakkers JT, Terwindt GM, Ferrari MD, Launer J (2004). Migraine as a risk factor for subclinical brain lesions. JAMA..

[53] Bashir A, Lipton RB, Ashina S, Ashina M (2013). Migraine and structural changes in the brain: a systematic review and meta-analysis. Neurology..

[54] Chong Catherine D, Schwedt Todd J, Hougaard Anders (2017). Brain functional connectivity in headache disorders: A narrative review of MRI investigations. Journal of Cerebral Blood Flow & Metabolism.

[55] Seitz I, Dirnagl U, Lindauer U (2004). Impaired vascular reactivity of isolated rat middle cerebral artery after cortical spreading depression in vivo. J Cereb Blood Flow Metab.

[56] Lauritzen M (1994). Pathophysiology of the migraine aura. The spreading depression theory. Brain..

[57] Thomsen LL, Iversen HK, Olesen J (1995). Increased cerebrovascular pCO2 reactivity in migraine with aura - a transcranial Doppler study during hyperventilation. Cephalalgia..

[58] Perko D, Pretnar-Oblak J, Sabovic M, Zvan B, Zaletel M (2011). Cerebrovascular reactivity to L-arginine in the anterior and posterior cerebral circulation in migraine patients. Acta Neurol Scand.

[59] Armulik A, Genové G, Betsholtz C (2011). Pericytes: developmental, physiological, and pathological perspectives, problems, and promises. Dev Cell.

[60] Hartmann DA, Underly RG, Grant RI, Watson AN, Lindner V, Shih AY (2015). Pericyte structure and distribution in the cerebral cortex revealed by high-resolution imaging of transgenic mice. Neurophotonics..

[61] Alarcon-Martinez L, Yilmaz-Ozcan S, Yemisci M, Schallek J, Kılıç K, Can A, Di Polo A, Dalkara T (2018). Capillary pericytes express α-smooth muscle actin, which requires prevention of filamentous-actin depolymerization for detection. Elife.

[62] Peppiatt CM, Howarth C, Mobbs P, Attwell D (2006). Bidirectional control of CNS capillary diameter by pericytes. Nature..

[63] Gursoy-Ozdemir Y, Yemisci M, Dalkara T (2012). Microvascular protection is essential for successful neuroprotection in stroke. J Neurochem.

[64] Montagne A, Barnes SR, Sweeney MD, Halliday MR, Sagare AP, Zhao Z, Toga AW, Jacobs RE, Liu CY, Amezcua L, Harrington MG, Chui HC, Law M, Zlokovic BV (2015). Blood-brain barrier breakdown in the aging human hippocampus. Neuron..

[65] Fernández-Klett F, Priller J (2015). Diverse functions of pericytes in cerebral blood flow regulation and ischemia. J Cereb Blood Flow Metab.

[66] Attwell D, Mishra A, Hall CN, O'Farrell FM, Dalkara T (2016). What is a pericyte?. J Cereb Blood Flow Metab.

[67] Kisler K, Nelson AR, Rege SV, Ramanathan A, Wang Y, Ahuja A, Lazic D, Tsai PS, Zhao Z, Zhou Y, Boas DA, Sakadžić S, Zlokovic BV (2017). Pericyte degeneration leads to neurovascular uncoupling and limits oxygen supply to brain. Nat Neurosci.

[68] Dalkara T, Alarcon-Martinez L, Yemisci M, Chen J, Zhang JH, Hu X (2016). Role of Pericytes in Neurovascular Unit and Stroke. Non-Neuronal Mechanisms of Brain Damage and Repair After Stroke.

[69] Yemisci M, Gursoy-Ozdemir Y, Vural A, Can A, Topalkara K, Dalkara T (2009). Pericyte contraction induced by oxidative-nitrative stress impairs capillary reflow despite successful opening of an occluded cerebral artery. Nat Med.

[70] Hall CN, Reynell C, Gesslein B, Hamilton NB, Mishra A, Sutherland BA, O'Farrell FM, Buchan AM, Lauritzen M, Attwell D (2014). Capillary pericytes regulate cerebral blood flow in health and disease. Nature.

[71] Khennouf L, Gesslein B, Brazhe A, Octeau JC, Kutuzov N, Khakh BS, Lauritzen M (2018). Active role of capillary pericytes during stimulation-induced activity and spreading depolarization. Brain..

[72] Joutel A, Corpechot C, Ducros A, Vahedi K, Chabriat H, Mouton P, Alamowitch S, Domenga V, Cécillion M, Marechal E, Maciazek J, Vayssiere C, Cruaud C, Cabanis EA, Ruchoux MM, Weissenbach J, Bach JF, Bousser MG, Tournier-Lasserve E (1996). Notch3 mutations in CADASIL, a hereditary adult-onset condition causing stroke and dementia. Nature..

[73] Dziewulska D, Lewandowska E (2012). Pericytes as a new target for pathological processes in CADASIL. Neuropathology..

[74] Gu X, Liu XY, Fagan A, Gonzalez-Toledo ME, Zhao LR (2012). Ultrastructural changes in cerebral capillary pericytes in aged Notch3 mutant transgenic mice. Ultrastruct Pathol.

[75] Ghosh M, Balbi M, Hellal F, Dichgans M, Lindauer U, Plesnila N (2015). Pericytes are involved in the pathogenesis of CADASIL. Ann Neurol.

[76] Chabriat H, Pappata S, Ostergaard L, Clark CA, Pachot-Clouard M, Vahedi K, Jobert A, Le Bihan D, Bousser MG (2000). Cerebral hemodynamics in cadasil before and after acetazolamide challenge assessed with MRI bolus tracking. Stroke..

[77] Bruening R, Dichgans M, Berchtenbreiter C, Yousry T, Seelos KC, Wu RH, Mayer M, Brix G, Reiser M (2001). Cerebral autosomal dominant arteriopathy with subcortical infarcts and leukoencephalopathy: decrease in regional cerebral blood volume in hyperintense subcortical lesions inversely correlates with disability and cognitive performance. AJNR Am J Neuroradiol.

[78] Pfefferkorn T, von Stuckrad-Barre S, Herzog J, Gasser T, Hamann GF, Dichgans M (2001). Reduced cerebrovascular CO_2_ reactivity in CADASIL: a transcranial doppler sonography study. Stroke..

[79] Ayata C (2010). CADASIL: experimental insights from animal models. Stroke..

[80] Dziewulska D, Kierdaszuk B (2018). Ultrastructural changes in microvessels in familial hemiplegic migraine with CACNA1A mutation. Clin Neuropathol.

[81] Cheng J, Korte N, Nortley R, Sethi H, Tang Y, Attwell D (2018). Targeting pericytes for therapeutic approaches to neurological disorders. Acta Neuropathol.

[82] Shin HK, Hong KW (2004). Importance of calcitonin gene-related peptide, adenosine and reactive oxygen species in cerebral autoregulation under normal and diseased conditions. Clin Exp Pharmacol Physiol.

[83] Zhai L, Sakurai T, Kamiyoshi A, Ichikawa-Shindo Y, Kawate H, Tanaka M, Xian X, Hirabayashi K, Dai K, Cui N, Tanimura K, Liu T, Wei Y, Tanaka M, Tomiyama H, Yamauchi A, Igarashi K, Shindo T (2018). Endogenous calcitonin gene-related peptide suppresses ischemic brain injuries and progression of cognitive decline. J Hypertens.

[84] Johansson SE, Abdolalizadeh B, Sheykhzade M, Edvinsson L, Sams A (2019). Vascular pathology of large cerebral arteries in experimental subarachnoid hemorrhage: vasoconstriction, functional CGRP depletion and maintained CGRP sensitivity. Eur J Pharmacol.

[85] Moskowitz MA, Reinhard JF, Romero J, Melamed E, Pettibone DJ (1979). Neurotransmitters and the fifth cranial nerve: is there a relation to the headache phase of migraine?. Lancet..

[86] Edvinsson L, Villalón CM, MaassenVanDenBrink A (2012). Basic mechanisms of migraine and its acute treatment. Pharmacol Ther.

[87] Goadsby PJ, Edvinsson L (1993). The trigeminovascular system and migraine: studies characterizing cerebrovascular and neuropeptide changes seen in humans and cats. Ann Neurol.

[88] Goadsby PJ, Edvinsson L, Ekman R (1988). Release of vasoactive peptides in the extracerebral circulation of humans and the cat during activation of the trigeminovascular system. Ann Neurol.

[89] Goadsby PJ, Edvinsson L, Ekman R (1990). Vasoactive peptide release in the extracerebral circulation of humans during migraine headache. Ann Neurol.

[90] Lassen LH, Haderslev PA, Jacobsen VB, Iversen HK, Sperling B, Olesen J (2002). CGRP may play a causative role in migraine. Cephalalgia..

[91] Deen M, Correnti E, Kamm K, Kelderman T, Papetti L, Rubio-Beltrán E, Vigneri S, Edvinsson L, MaassenVanDenBrink A (2017). European Headache Federation School of Advanced Studies (EHF-SAS). Blocking CGRP in migraine patients - a review of pros and cons. J Headache Pain.

[92] MaassenVanDenBrink A, Meijer J, Villalón CM, Ferrari MD (2016). Wiping out CGRP: potential cardiovascular risks. Trends Pharmacol Sci.

[93] Favoni V, Giani L, Al-Hassany L, Asioli GM, Butera C, de Boer I, Guglielmetti M, Koniari C, Mavridis T, Vaikjärv M, Verhagen I, Verzina A, Zick B, Martelletti P, Sacco S, European Headache Federation School of Advanced Studies (EHF-SAS) (2019). CGRP and migraine from a cardiovascular point of view: what do we expect from blocking CGRP?. J Headache Pain.

[94] Moskowitz MA, Nozaki K, Kraig RP (1993). Neocortical spreading depression provokes the expression of c-fos protein-like immunoreactivity within trigeminal nucleus caudalis via trigeminovascular mechanisms. J Neurosci.

[95] Bolay H, Reuter U, Dunn AK, Huang Z, Boas DA, Moskowitz MA (2002). Intrinsic brain activity triggers trigeminal meningeal afferents in a migraine model. Nat Med.

[96] Karatas H, Erdener SE, Gursoy-Ozdemir Y, Lule S, Eren-Kocak E, Sen ZD, Dalkara T (2013). Spreading depression triggers headache by activating neuronal Panx1 channels. Science..

[97] Chen SP, Qin T, Seidel JL, Zheng Y, Eikermann M, Ferrari MD, van den Maagdenberg AMJM, Moskowitz MA, Ayata C, Eikermann-Haerter K (2017). Inhibition of the P2X7-PANX1 complex suppresses spreading depolarization and neuroinflammation. Brain..

[98] Yamasaki Y, Matsuura N, Shozuhara H, Onodera H, Itoyama Y, Kogure K (1995). Interleukin-1 as a pathogenetic mediator of ischemic brain damage in rats. Stroke..

[99] Neeb L, Hellen P, Boehnke C, Hoffmann J, Schuh-Hofer S, Dirnagl U (2011). IL-1beta stimulates COX-2 dependent PGE (2) synthesis and CGRP release in rat trigeminal ganglia cells. PLoS One.

[100] Lambertsen KL, Biber K, Finsen B (2012). Inflammatory cytokines in experimental and human stroke. J Cereb Blood Flow Metab.

[101] Zhang X, Burstein R, Levy D (2012). Local action of the proinflammatory cytokines IL-1beta and IL-6 on intracranial meningeal nociceptors. Cephalalgia..

[102] Orr SL, Dos Santos MP, Jurencak R, Michaud J, Miller E, Doja A (2014). Central nervous system venulitis presenting as migraine. Headache..

[103] Sakadzic S, Mandeville ET, Gagnon L, Musacchia JJ, Yaseen MA, Yucel MA, Lefebvre J, Lesage F, Dale AM, Eikermann-Haerter K, Ayata C, Srinivasan VJ, Lo EH, Devor A, Boas DA (2014). Large arteriolar component of oxygen delivery implies a safe margin of oxygen supply to cerebral tissue. Nat Commun.

[104] Dönmez-Demir B, Yemisci M, Dalkara T (2018). Data of ascending cortical vein occlusion induced spreading depression. Data Brief.

[105] Snijder RJ, Luermans JG, de Heij AH, Thijs V, Schonewille WJ, Van De Bruaene A, Swaans MJ, Budts WI, Post MC (2016) Patent Foramen Ovale With Atrial Septal Aneurysm Is Strongly Associated With Migraine With Aura: A Large Observational Study. J Am Heart Assoc 5(12):e003771 10.1161/JAHA.116.003771 PMC5210450 27930349

[106] Noheria A, Roshan J, Kapa S, Srivathsan K, Packer DL, Asirvatham SJ (2011). Migraine headaches following catheter ablation for atrial fibrillation. J Interv Card Electrophysiol.

[107] Sevgi EB, Erdener SE, Demirci M, Topcuoglu MA, Dalkara T (2012) Paradoxical air microembolism induces cerebral bioelectrical abnormalities and occasionally headache in patent foramen ovale patients with migraine. J Am Heart Assoc 1(6):e001735 10.1161/JAHA.112.001735 PMC3540661 23316313

[108] Buture A, Khalil M, Ahmed F (2017). Iatrogenic visual aura: a case report and a brief review of the literature. Ther Clin Risk Manag.

[109] Androulakis XM, Kodumuri N, Giamberardino LD, Rosamond WD, Gottesman RF, Yim E, Sen S (2016). Ischemic stroke subtypes and migraine with visual aura in the ARIC study. Neurology..

[110] Timm FP, Houle TT, Grabitz SD, Lihn A, Stokholm JB, Eikermann-Haerter K, Nozari A, Kurth T, Eikermann M (2017). Migraine and risk of perioperative ischemic stroke and hospital readmission: hospital based registry study. BMJ..

